# Supplementary material for: Red Blood Cell BCL-xL Is Required for Plasmodium falciparum Survival: Insights into Host-Directed Malaria Therapies
Source: Microorganisms. 2022 Apr 15;10(4):824. doi: 10.3390/microorganisms10040824 (PMC9027239; doi:10.3390/microorganisms10040824)
Supplement: Supplementary file 1 [file microorganisms-10-00824-s001.zip › microorganisms-1678255-supplementary.pptx]

## Slide 1
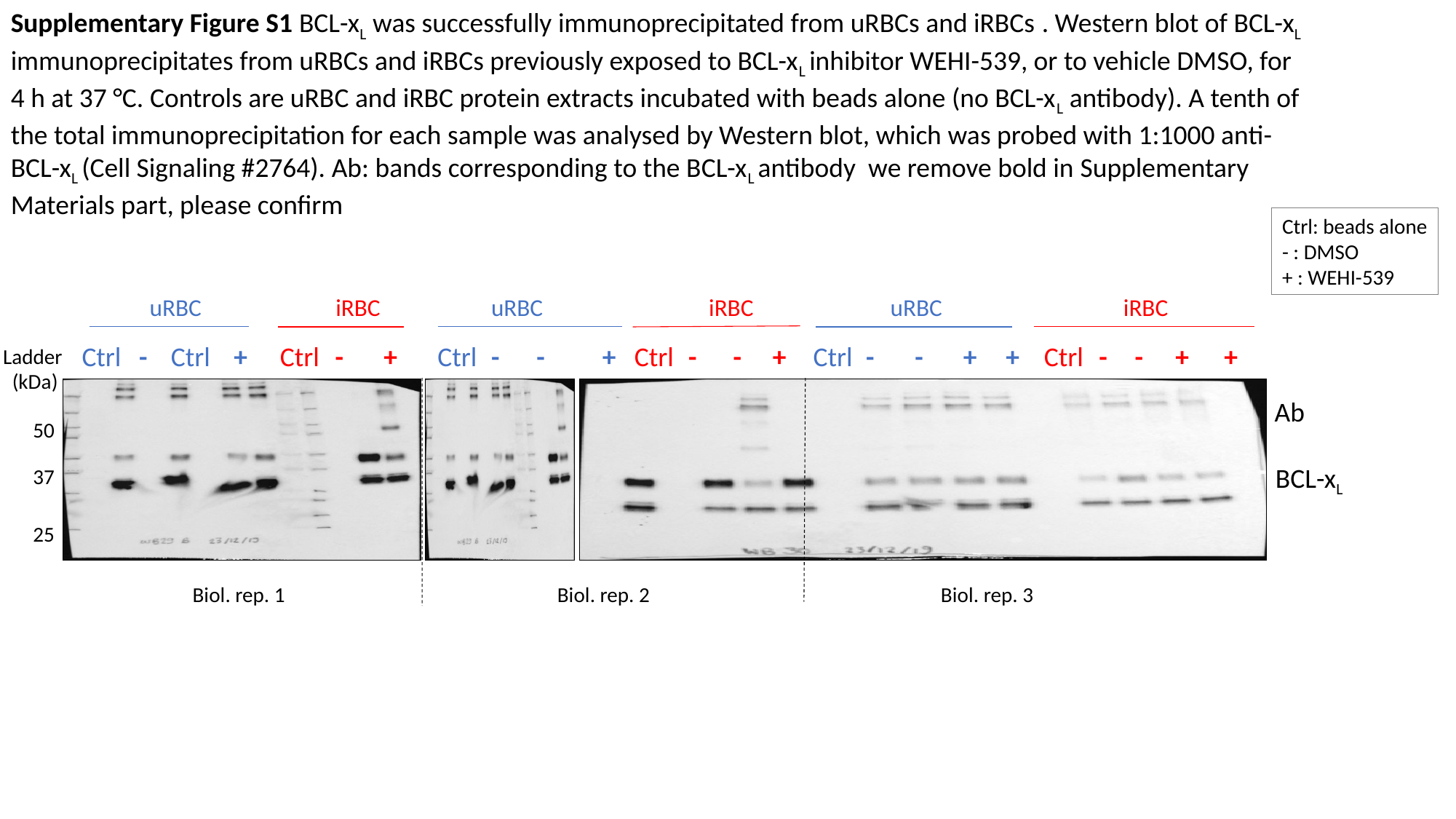

Supplementary Figure S1 BCL-xL was successfully immunoprecipitated from uRBCs and iRBCs . Western blot of BCL-xL immunoprecipitates from uRBCs and iRBCs previously exposed to BCL-xL inhibitor WEHI-539, or to vehicle DMSO, for 4 h at 37 °C. Controls are uRBC and iRBC protein extracts incubated with beads alone (no BCL-xL antibody). A tenth of the total immunoprecipitation for each sample was analysed by Western blot, which was probed with 1:1000 anti-BCL-xL (Cell Signaling #2764). Ab: bands corresponding to the BCL-xL antibody  we remove bold in Supplementary Materials part, please confirm
Ctrl: beads alone
- : DMSO
+ : WEHI-539
uRBC
iRBC
uRBC
iRBC
uRBC
iRBC
-
+
-
+
-
-
+
-
-
+
-
-
+
+
-
-
+
+
Ctrl
Ctrl
Ctrl
Ctrl
Ctrl
Ctrl
Ctrl
Ladder
(kDa)
Ab
50
BCL-xL
37
25
Biol. rep. 1
Biol. rep. 2
Biol. rep. 3

## Slide 2
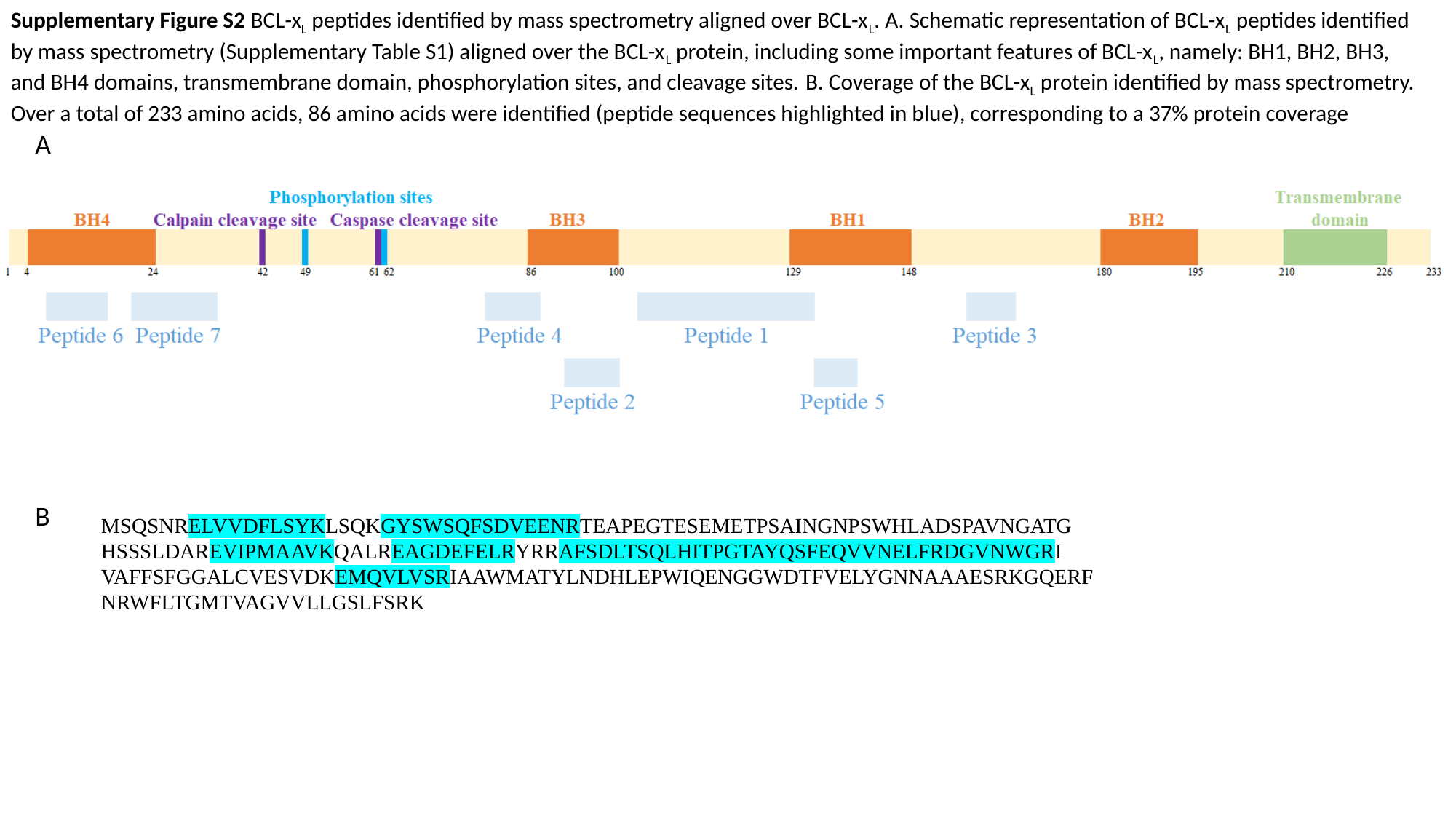

Supplementary Figure S2 BCL-xL peptides identified by mass spectrometry aligned over BCL-xL. A. Schematic representation of BCL-xL peptides identified by mass spectrometry (Supplementary Table S1) aligned over the BCL-xL protein, including some important features of BCL-xL, namely: BH1, BH2, BH3, and BH4 domains, transmembrane domain, phosphorylation sites, and cleavage sites. B. Coverage of the BCL-xL protein identified by mass spectrometry. Over a total of 233 amino acids, 86 amino acids were identified (peptide sequences highlighted in blue), corresponding to a 37% protein coverage
A
B
MSQSNRELVVDFLSYKLSQKGYSWSQFSDVEENRTEAPEGTESEMETPSAINGNPSWHLADSPAVNGATG
HSSSLDAREVIPMAAVKQALREAGDEFELRYRRAFSDLTSQLHITPGTAYQSFEQVVNELFRDGVNWGRI
VAFFSFGGALCVESVDKEMQVLVSRIAAWMATYLNDHLEPWIQENGGWDTFVELYGNNAAAESRKGQERF
NRWFLTGMTVAGVVLLGSLFSRK

## Slide 3
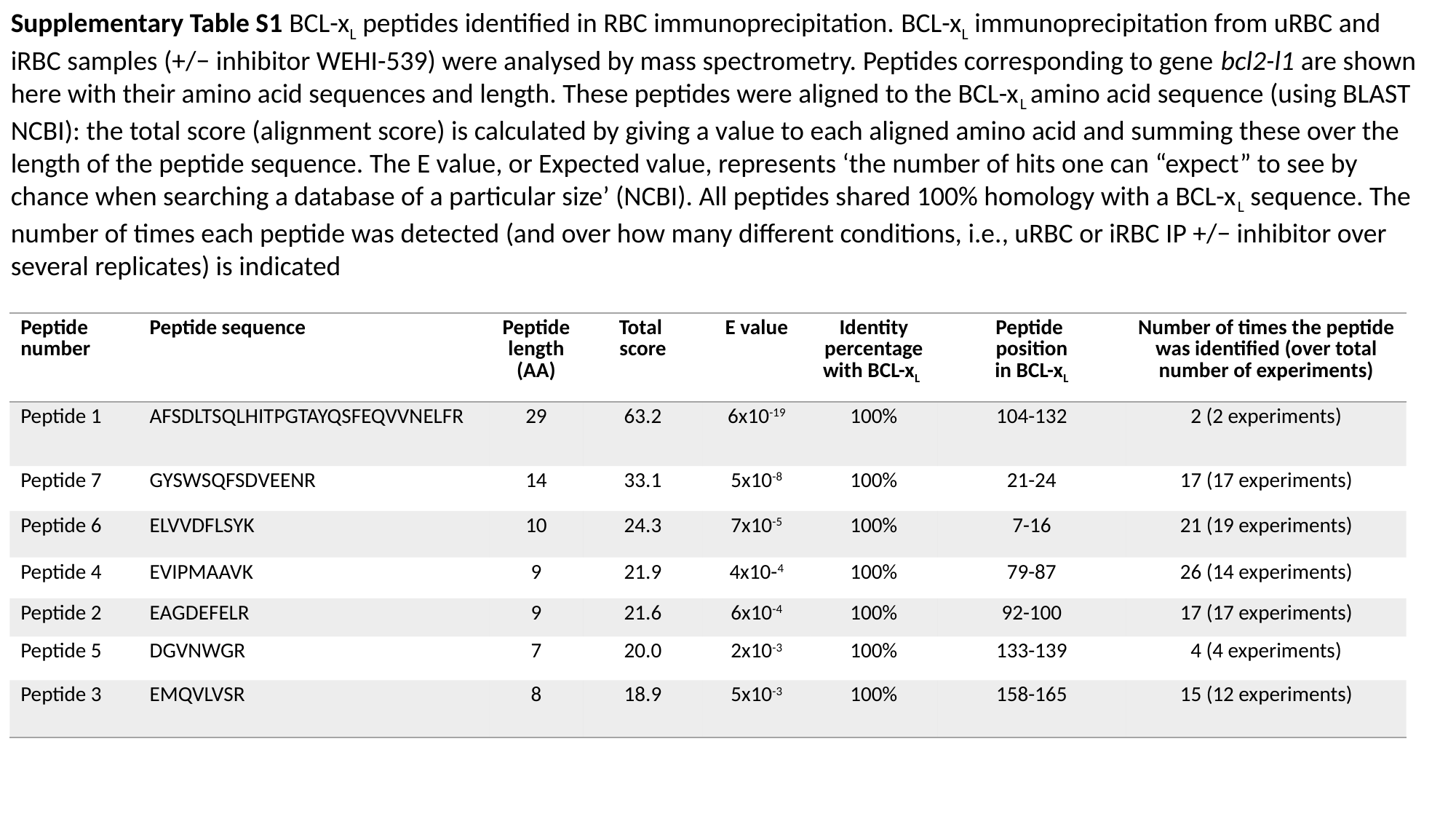

Supplementary Table S1 BCL-xL peptides identified in RBC immunoprecipitation. BCL-xL immunoprecipitation from uRBC and iRBC samples (+/− inhibitor WEHI-539) were analysed by mass spectrometry. Peptides corresponding to gene bcl2-l1 are shown here with their amino acid sequences and length. These peptides were aligned to the BCL-xL amino acid sequence (using BLAST NCBI): the total score (alignment score) is calculated by giving a value to each aligned amino acid and summing these over the length of the peptide sequence. The E value, or Expected value, represents ‘the number of hits one can “expect” to see by chance when searching a database of a particular size’ (NCBI). All peptides shared 100% homology with a BCL-xL sequence. The number of times each peptide was detected (and over how many different conditions, i.e., uRBC or iRBC IP +/− inhibitor over several replicates) is indicated
| Peptide number | Peptide sequence | Peptide length (AA) | Total score | E value | Identity percentage with BCL-xL | Peptide position in BCL-xL | Number of times the peptide was identified (over total number of experiments) |
| --- | --- | --- | --- | --- | --- | --- | --- |
| Peptide 1 | AFSDLTSQLHITPGTAYQSFEQVVNELFR | 29 | 63.2 | 6x10^-19^ | 100% | 104-132 | 2 (2 experiments) |
| Peptide 7 | GYSWSQFSDVEENR | 14 | 33.1 | 5x10^-8^ | 100% | 21-24 | 17 (17 experiments) |
| Peptide 6 | ELVVDFLSYK | 10 | 24.3 | 7x10^-5^ | 100% | 7-16 | 21 (19 experiments) |
| Peptide 4 | EVIPMAAVK | 9 | 21.9 | 4x10^-4^ | 100% | 79-87 | 26 (14 experiments) |
| Peptide 2 | EAGDEFELR | 9 | 21.6 | 6x10^-4^ | 100% | 92-100 | 17 (17 experiments) |
| Peptide 5 | DGVNWGR | 7 | 20.0 | 2x10^-3^ | 100% | 133-139 | 4 (4 experiments) |
| Peptide 3 | EMQVLVSR | 8 | 18.9 | 5x10^-3^ | 100% | 158-165 | 15 (12 experiments) |

## Slide 4
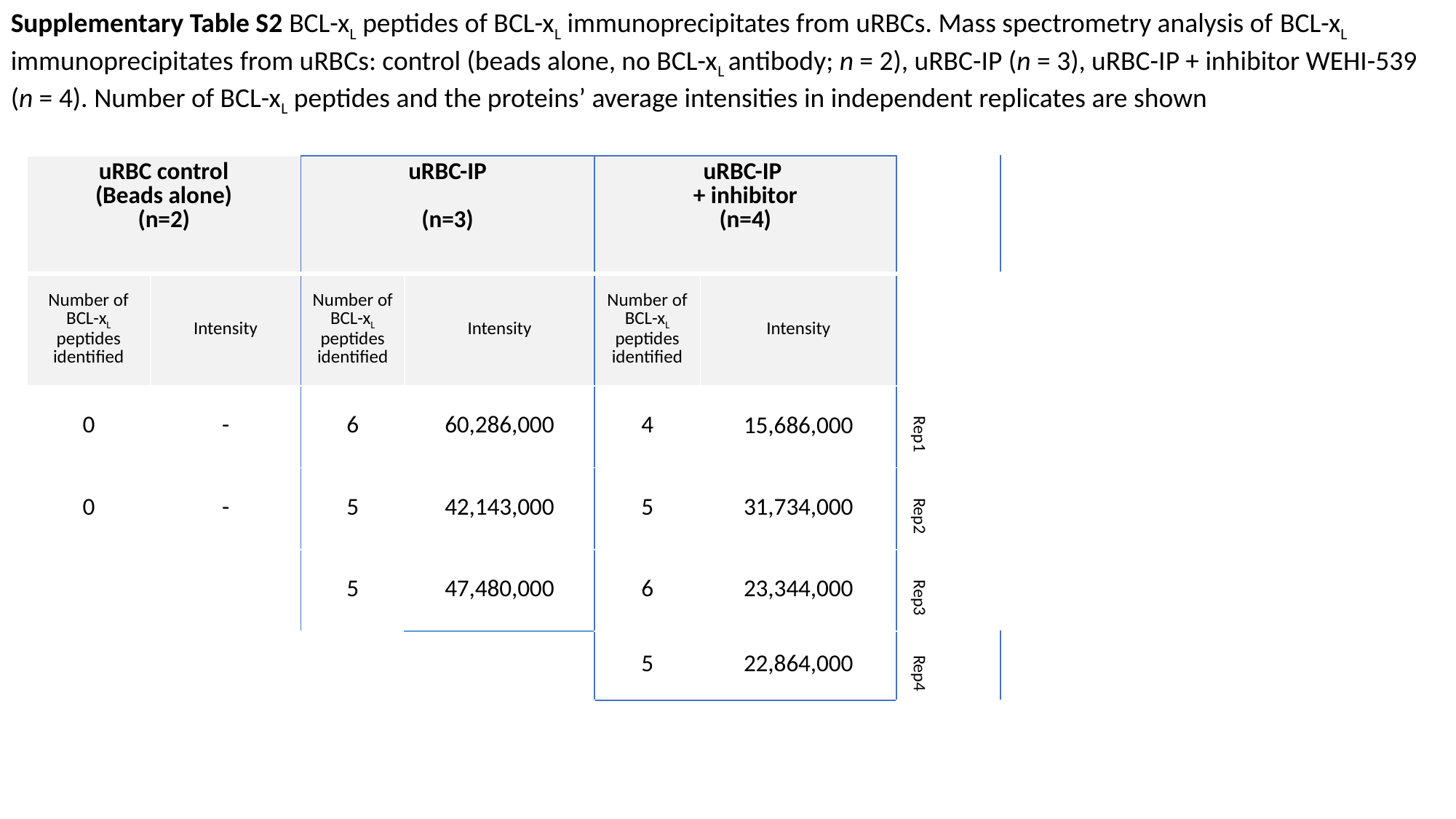

Supplementary Table S2 BCL-xL peptides of BCL-xL immunoprecipitates from uRBCs. Mass spectrometry analysis of BCL-xL immunoprecipitates from uRBCs: control (beads alone, no BCL-xL antibody; n = 2), uRBC-IP (n = 3), uRBC-IP + inhibitor WEHI-539 (n = 4). Number of BCL-xL peptides and the proteins’ average intensities in independent replicates are shown
| uRBC control (Beads alone) (n=2) | | uRBC-IP (n=3) | | uRBC-IP + inhibitor (n=4) | | |
| --- | --- | --- | --- | --- | --- | --- |
| Number of BCL-xL peptides identified | Intensity | Number of BCL-xL peptides identified | Intensity | Number of BCL-xL peptides identified | Intensity | |
| 0 | - | 6 | 60,286,000 | 4 | 15,686,000 | Rep1 |
| 0 | - | 5 | 42,143,000 | 5 | 31,734,000 | Rep2 |
| | | 5 | 47,480,000 | 6 | 23,344,000 | Rep3 |
| | | | | 5 | 22,864,000 | Rep4 |
| | | | | | | |

## Slide 5
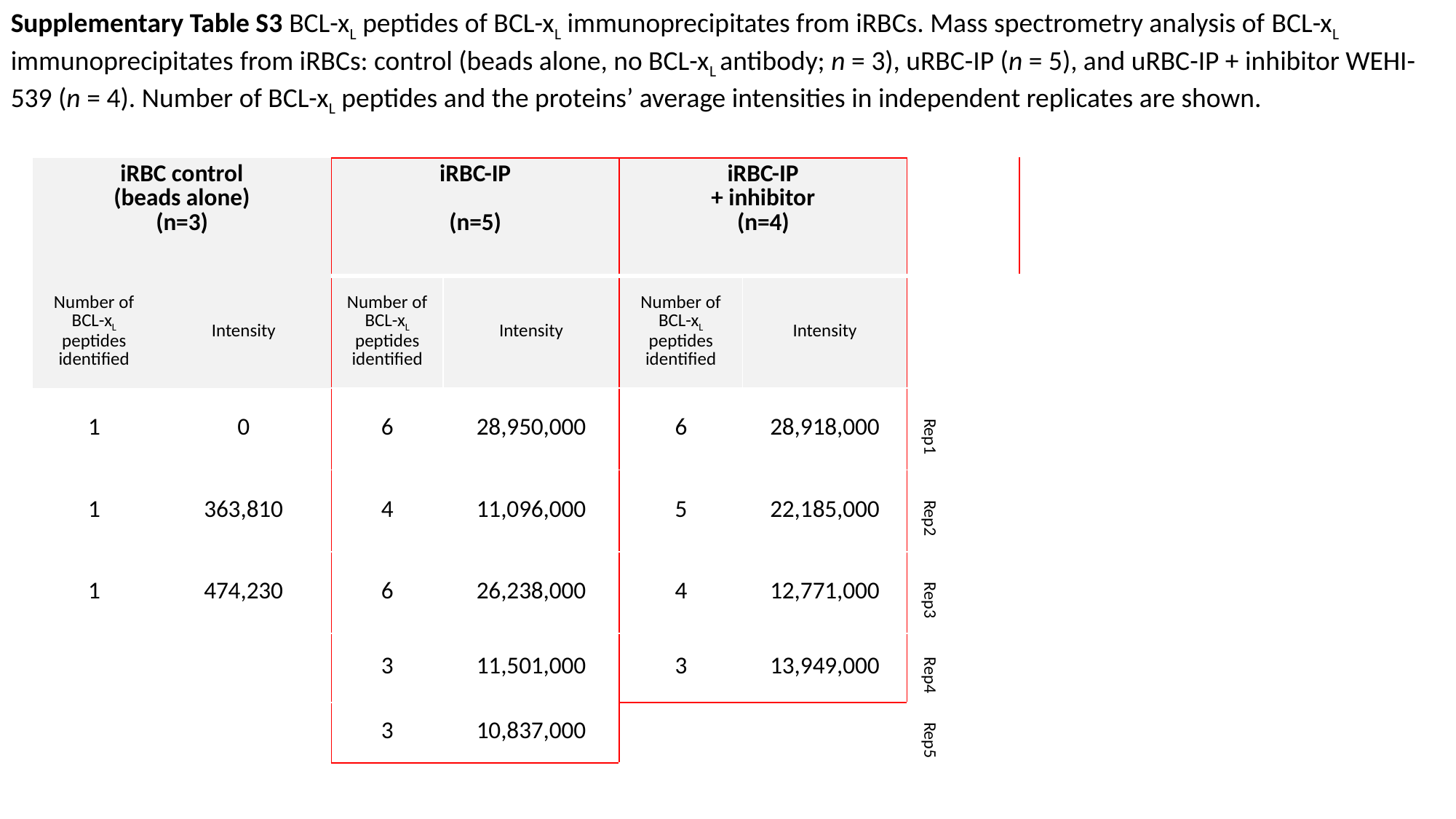

Supplementary Table S3 BCL-xL peptides of BCL-xL immunoprecipitates from iRBCs. Mass spectrometry analysis of BCL-xL immunoprecipitates from iRBCs: control (beads alone, no BCL-xL antibody; n = 3), uRBC-IP (n = 5), and uRBC-IP + inhibitor WEHI-539 (n = 4). Number of BCL-xL peptides and the proteins’ average intensities in independent replicates are shown.
| iRBC control (beads alone) (n=3) | | iRBC-IP (n=5) | | iRBC-IP + inhibitor (n=4) | | |
| --- | --- | --- | --- | --- | --- | --- |
| Number of BCL-xL peptides identified | Intensity | Number of BCL-xL peptides identified | Intensity | Number of BCL-xL peptides identified | Intensity | |
| 1 | 0 | 6 | 28,950,000 | 6 | 28,918,000 | Rep1 |
| 1 | 363,810 | 4 | 11,096,000 | 5 | 22,185,000 | Rep2 |
| 1 | 474,230 | 6 | 26,238,000 | 4 | 12,771,000 | Rep3 |
| | | 3 | 11,501,000 | 3 | 13,949,000 | Rep4 |
| | | 3 | 10,837,000 | | | Rep5 |

## Slide 6
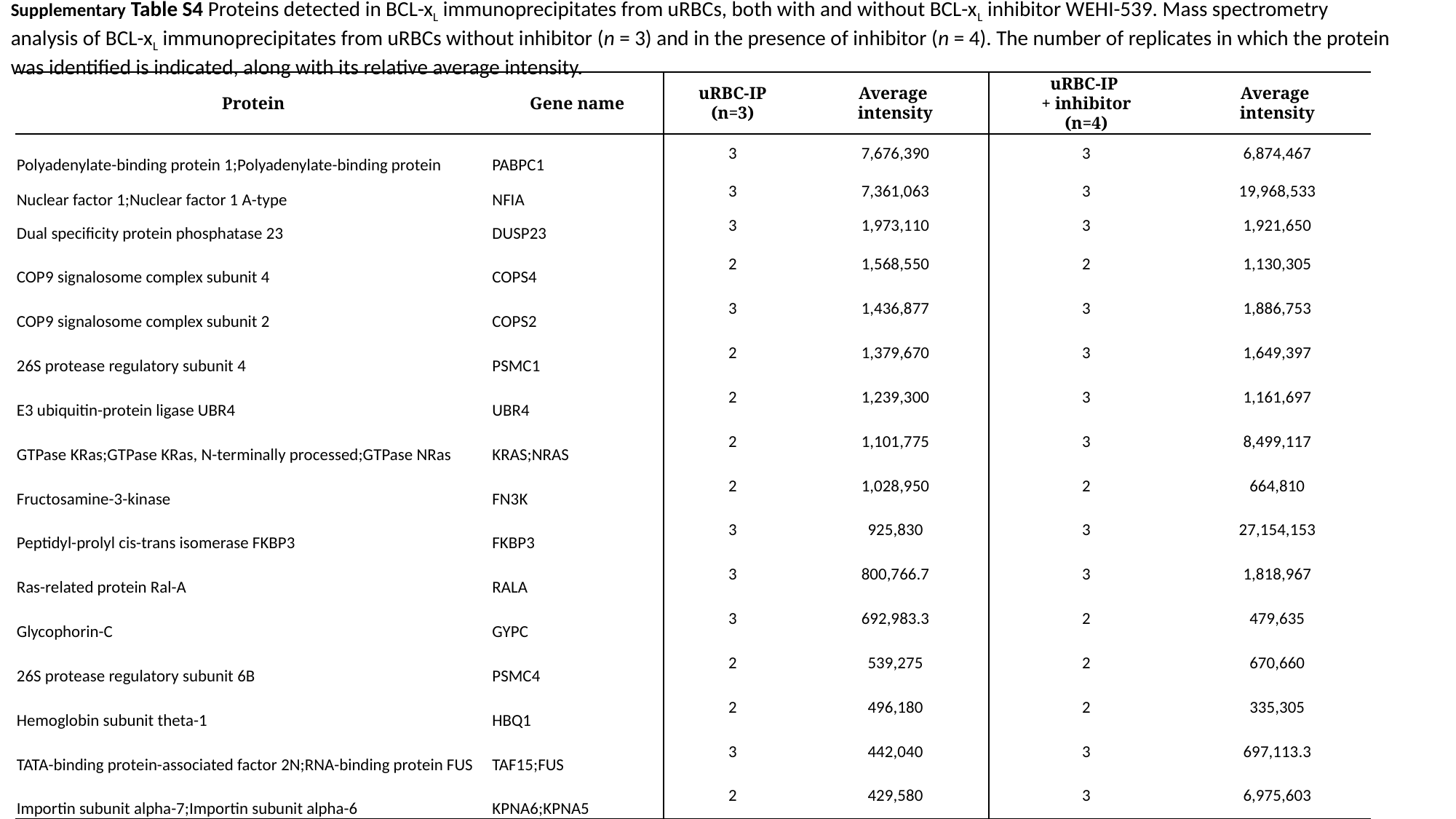

Supplementary Table S4 Proteins detected in BCL-xL immunoprecipitates from uRBCs, both with and without BCL-xL inhibitor WEHI-539. Mass spectrometry analysis of BCL-xL immunoprecipitates from uRBCs without inhibitor (n = 3) and in the presence of inhibitor (n = 4). The number of replicates in which the protein was identified is indicated, along with its relative average intensity.
| Protein | Gene name | uRBC-IP (n=3) | Average intensity | uRBC-IP + inhibitor (n=4) | Average intensity |
| --- | --- | --- | --- | --- | --- |
| Polyadenylate-binding protein 1;Polyadenylate-binding protein | PABPC1 | 3 | 7,676,390 | 3 | 6,874,467 |
| Nuclear factor 1;Nuclear factor 1 A-type | NFIA | 3 | 7,361,063 | 3 | 19,968,533 |
| Dual specificity protein phosphatase 23 | DUSP23 | 3 | 1,973,110 | 3 | 1,921,650 |
| COP9 signalosome complex subunit 4 | COPS4 | 2 | 1,568,550 | 2 | 1,130,305 |
| COP9 signalosome complex subunit 2 | COPS2 | 3 | 1,436,877 | 3 | 1,886,753 |
| 26S protease regulatory subunit 4 | PSMC1 | 2 | 1,379,670 | 3 | 1,649,397 |
| E3 ubiquitin-protein ligase UBR4 | UBR4 | 2 | 1,239,300 | 3 | 1,161,697 |
| GTPase KRas;GTPase KRas, N-terminally processed;GTPase NRas | KRAS;NRAS | 2 | 1,101,775 | 3 | 8,499,117 |
| Fructosamine-3-kinase | FN3K | 2 | 1,028,950 | 2 | 664,810 |
| Peptidyl-prolyl cis-trans isomerase FKBP3 | FKBP3 | 3 | 925,830 | 3 | 27,154,153 |
| Ras-related protein Ral-A | RALA | 3 | 800,766.7 | 3 | 1,818,967 |
| Glycophorin-C | GYPC | 3 | 692,983.3 | 2 | 479,635 |
| 26S protease regulatory subunit 6B | PSMC4 | 2 | 539,275 | 2 | 670,660 |
| Hemoglobin subunit theta-1 | HBQ1 | 2 | 496,180 | 2 | 335,305 |
| TATA-binding protein-associated factor 2N;RNA-binding protein FUS | TAF15;FUS | 3 | 442,040 | 3 | 697,113.3 |
| Importin subunit alpha-7;Importin subunit alpha-6 | KPNA6;KPNA5 | 2 | 429,580 | 3 | 6,975,603 |

## Slide 7
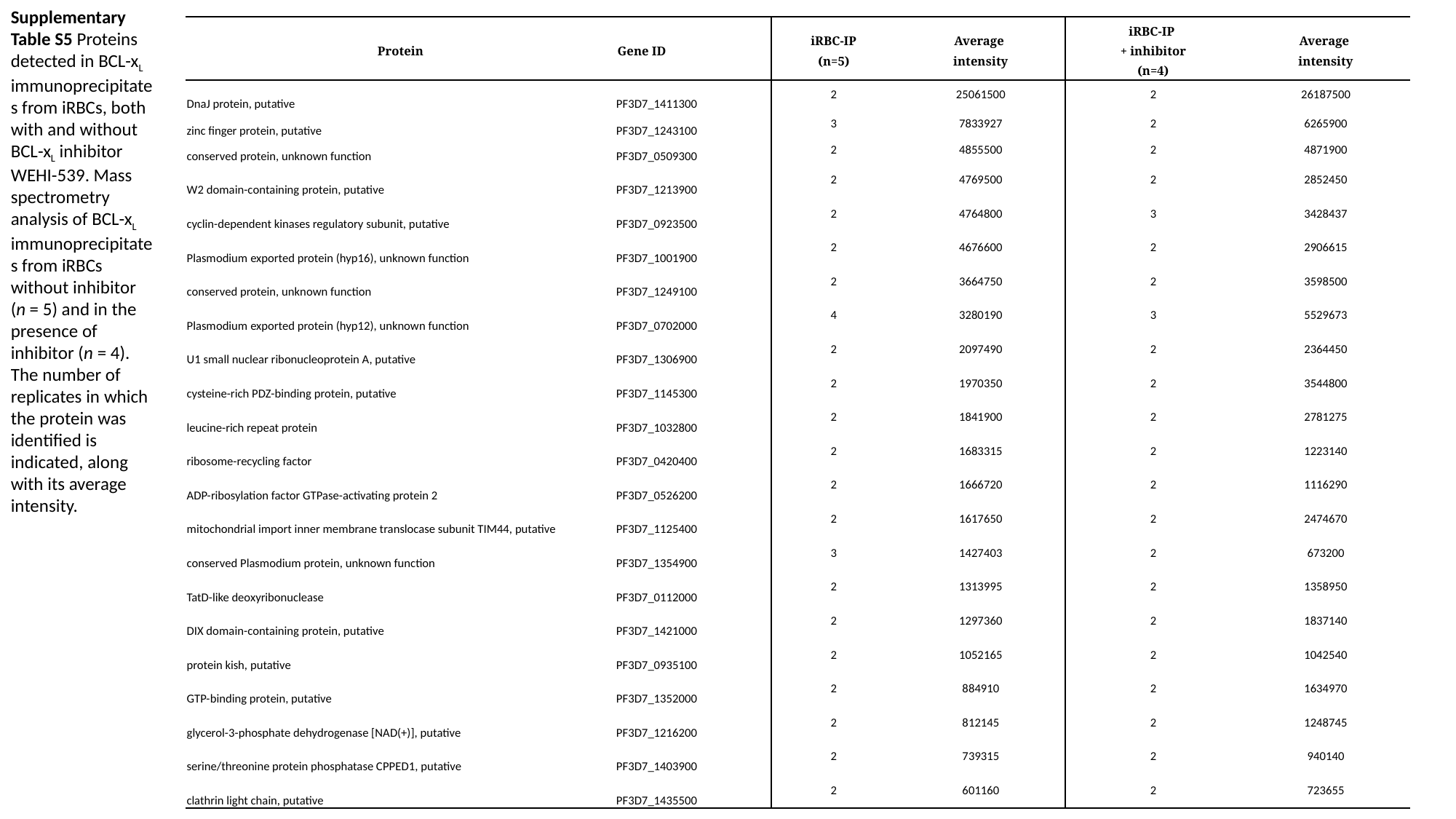

Supplementary Table S5 Proteins detected in BCL-xL immunoprecipitates from iRBCs, both with and without BCL-xL inhibitor WEHI-539. Mass spectrometry analysis of BCL-xL immunoprecipitates from iRBCs without inhibitor (n = 5) and in the presence of inhibitor (n = 4). The number of replicates in which the protein was identified is indicated, along with its average intensity.
| Protein | Gene ID | iRBC-IP (n=5) | Average intensity | iRBC-IP + inhibitor (n=4) | Average intensity |
| --- | --- | --- | --- | --- | --- |
| DnaJ protein, putative | PF3D7\_1411300 | 2 | 25061500 | 2 | 26187500 |
| zinc finger protein, putative | PF3D7\_1243100 | 3 | 7833927 | 2 | 6265900 |
| conserved protein, unknown function | PF3D7\_0509300 | 2 | 4855500 | 2 | 4871900 |
| W2 domain-containing protein, putative | PF3D7\_1213900 | 2 | 4769500 | 2 | 2852450 |
| cyclin-dependent kinases regulatory subunit, putative | PF3D7\_0923500 | 2 | 4764800 | 3 | 3428437 |
| Plasmodium exported protein (hyp16), unknown function | PF3D7\_1001900 | 2 | 4676600 | 2 | 2906615 |
| conserved protein, unknown function | PF3D7\_1249100 | 2 | 3664750 | 2 | 3598500 |
| Plasmodium exported protein (hyp12), unknown function | PF3D7\_0702000 | 4 | 3280190 | 3 | 5529673 |
| U1 small nuclear ribonucleoprotein A, putative | PF3D7\_1306900 | 2 | 2097490 | 2 | 2364450 |
| cysteine-rich PDZ-binding protein, putative | PF3D7\_1145300 | 2 | 1970350 | 2 | 3544800 |
| leucine-rich repeat protein | PF3D7\_1032800 | 2 | 1841900 | 2 | 2781275 |
| ribosome-recycling factor | PF3D7\_0420400 | 2 | 1683315 | 2 | 1223140 |
| ADP-ribosylation factor GTPase-activating protein 2 | PF3D7\_0526200 | 2 | 1666720 | 2 | 1116290 |
| mitochondrial import inner membrane translocase subunit TIM44, putative | PF3D7\_1125400 | 2 | 1617650 | 2 | 2474670 |
| conserved Plasmodium protein, unknown function | PF3D7\_1354900 | 3 | 1427403 | 2 | 673200 |
| TatD-like deoxyribonuclease | PF3D7\_0112000 | 2 | 1313995 | 2 | 1358950 |
| DIX domain-containing protein, putative | PF3D7\_1421000 | 2 | 1297360 | 2 | 1837140 |
| protein kish, putative | PF3D7\_0935100 | 2 | 1052165 | 2 | 1042540 |
| GTP-binding protein, putative | PF3D7\_1352000 | 2 | 884910 | 2 | 1634970 |
| glycerol-3-phosphate dehydrogenase [NAD(+)], putative | PF3D7\_1216200 | 2 | 812145 | 2 | 1248745 |
| serine/threonine protein phosphatase CPPED1, putative | PF3D7\_1403900 | 2 | 739315 | 2 | 940140 |
| clathrin light chain, putative | PF3D7\_1435500 | 2 | 601160 | 2 | 723655 |
